# Narrowband-Internet of Things Device-to-Device Simulation: An Open-Sourced Framework

**DOI:** 10.3390/s21051824

**Published:** 2021-03-05

**Authors:** Ohood Saud Althobaiti, Mischa Dohler

**Affiliations:** Department of Engineering, King’s College London, London, WC2R 2LS, UK; mischa.dohler@kcl.ac.uk

**Keywords:** narrow band, Internet of Things, simulation, simulator, path loss, energy consumption, power profile, adaptive clustering hierarchy, channel scheduling, device-to-device (D2D) communication

## Abstract

Narrowband-Internet of Things (NB-IoT) displays high-quality connectivity underpinned by fifth-generation (5G) networks to cover a wide array of IoT applications. The devices’ development and integration into different smart systems require permanent control, supervision, and the study of power consumption models to determine the performance of the network topology and allow for the measurement of the efficiency of the network topology’s application. This paper reports on an architecture and open-sourced simulation that was developed to study NB-IoT in Device-to-Device (D2D) mode, which includes the Physical (PHY), network, and application layers, as well as a queuing model, the model for uplink and downlink delays, the throughput, the overall NB-IoT D2D network performance, and the energy consumption based on the Low Energy Adaptive Clustering Hierarchy (LEACH) protocol. Our results prove that the suggested framework contributes to a reduction in power consumption, a minimization of queuing delays, a decrease in communication cost, a reduction in inter-cluster collisions, and the prevention of attacks from malicious nodes. Consequently, the framework manages the battery’s State of Charge (SOC), improves the battery’s State of Health (SOH), and maximizes the whole network lifetime. The proposed framework, the code of which has been open-sourced, can be effectively used for scientific research and development purposes to evaluate different parameters and improve the planning of NB-IoT networks.

## 1. Introduction

The Internet of Things (IoT) is as an emerging technology that can connect trillions of embedded devices at once. This enables innovative applications in almost every sector. Examples are intelligent connected vehicles, monitoring systems that share real-time information, unmanned aerial vehicles, the smart grid, smart homes, smart cities, and smart farming. The IoT is changing the world of logistics, construction, healthcare, operation and maintenance of infrastructure, and a lot more. The IoT has increasingly gained research awareness with its ability to forecast, monitor, prevent, and track infectious disease epidemics. IoT-based health management schemes offer instantaneous observation from wearable appliances and Artificial Intelligence (AI), including neural networks, deep learning algorithms, and remote tests through cloud computing to conduct health investigations [1]. As a result, medical staff can detect the coronavirus disease-2019 (COVID-19) without jeopardizing their lives or causing the virus to spread to other locations [2]. IoT-based healthcare systems can be an important advancement in the measures to combat the current pandemic [1].

For IoT connectivity, there are different communication technologies to connect the actuators and sensors. Wireless Fidelity (Wi-Fi, based on IEEE 802.11) networking is one of the most widely used protocols for connectivity. Users’ equipment can access the network through a single access point. The topologies in Wi-Fi involve peer-to-peer and star topologies with an expanded coverage of up to 100 m. The topology of peer-to-peer connectivity is compatible with an advanced feature of 5G mobile peer-to-peer connections, notably Device-to-Device (D2D) communications. This has allowed emergency systems based on IoT to collect and send information automatically and reliably over the Internet [3]. In addition, Wi-Fi (IEEE 802.11 AC) is a 2.4 to 5 GHz band system. Due to its geographic range and high data rate, Wi-Fi infrastructure yields high energy consumption. Therefore, it is typically used in situations that need high data rates such as wireless monitoring cameras [3]. Bluetooth Low Energy (BLE) has also helped IoT to grow rapidly [4].

The number of nodes that can be supported by Wi-Fi or BLE depends on operational circumstances. However, the number of devices on Wi-Fi can reach up to 250, depending on the capabilities of the access points [5]. On BLE, up to seven devices can be linked simultaneously and can exchange data using the BLE standard. The main performance metrics that Wi-Fi can achieve involve performing at a high data rate, improving the scalability of the number of nodes, protecting user identity, mobility, reliability, and zero complexity. However, long battery life has not been achieved successfully with Wi-Fi, whilst long range has not been achieved with BLE. Neither of the two can ensure a guaranteed Quality of Service (QoS), which is because of the use of license-exempt spectrum.

These embedded connectivity technologies have been deployed over short ranges, but they have proven to be unsuccessful at meeting the escalating demands of IoT devices. These principally require technologies that consume low energy and long communications range [6,7]. As a result, Low-Power Wide Area Network (LPWAN) technologies have arisen. The most advanced LPWAN systems providing large-scale coverage are Narrowband-Internet of Things (NB-IoT), (to some extent) Long Term Evolution for Machines (LTE-M), LoRa, and SigFox. Cellular networks have been implemented to provide larger connectivity coverage, but because of the high energy usage on nodes, they were not as effective [6,7]. Furthermore, the growing use of LPWANs in industry and the research community is driven by the long-range, low cost, and low-energy functionalities of these systems. However, NB-IoT introduces a guaranteed QoS when compared wtih SigFox or LoRa due to the use of licensed spectrum. QoS assurance is an important concern for industrial IoT. Moreover, the energy efficiency of NB-IoT is significantly higher than in LTE-M. The battery life of a device can be more than 10 years in the case of NB-IoT, but it is only 5–10 years in LTE-M. Maximum data rates for NB-IoT, SigFox, and LoRa are 250 kbps, 100 bps, and 50 kbps, respectively. In addition, NB-IoT provides lower latency compared to SigFox and LoRa.

The authors in [8] argue that because of new developments to miniaturize devices while maintaining higher processing capabilities and low-power networking, it possible for IoT to be deployed widely everywhere. Unfortunately, these advancements require modifications in network structure, software, hardware design, energy sources, and data management. Since regular maintenance and replacement of batteries are unlikely in locations that are difficult to access, IoT devices must consume ultra-low energy so as to last for years. Hence, NB-IoT is a promising solution and is indeed becoming increasingly common as it is extremely energy efficient, whilst offering operational advantages as discussed above.

In this paper, we prove that NB-IoT can be power optimized further with the latest 3rd Generation Partnership Project (3GPP) tools. These include Device-to-Device (D2D) communications, which is a promising solution for extending the battery life of IoT devices. D2D connectivity between devices near to each other is characterized as direct data traffic. It offers significant promise to enhance power effectiveness, spectrum performance, and throughput, while it minimizes latency and maintains QoS assurances [9,10]. The recovery of local area networking and the development of spectrum performance are two core issues of LTE-Advanced. D2D communication, which provides mobile peer-to-peer connectivity, can solve these issues [10]. Initially, D2D connectivity was suggested as a new model for optimizing network efficiency in cellular networks [10].

The main “selling point” of NB-IoT is its low energy consumption which increases the battery life time of embedded devices [11,12,13]. This study considers the NB-IoT’s energy consumption, throughput, and performance model to determine the power profile achieved in an attocell IoT network formed by D2D links. Findings are validated through a developed simulator that uses a Graphical User Interface (GUI) for the NB-IoT protocol with a stack that operates in up and downlinks, multiple users, Multiple-Input Multiple-Output (MIMO) PHY arrays, a Low Energy Adaptive Clustering Hierarchy (LEACH) protocol, Adaptive Modulation and Coding (AMC), and scheduling procedures. This framework is proposed to help reduce the energy consumption of NB-IoT further, and thus aid the rollout of billions of connections over the coming years.

The design of the NB-IoT physical layer inherits the main features of LTE. Empirical models are typically used to predict the propagation paradigms for LTE and LTE-Advanced networks [14]. However, NB-IoT presents three physical channels and three signaling channels for downlink: Narrowband Physical Downlink Control Channel (NPDCCH), Narrowband Physical Broadcast Channel (NPBCH), Narrowband Physical Downlink Shared Channel (NPDSCH), Narrowband Primary Synchronization Signal (NPSS), Narrowband Reference Signal (NRS) and Narrowband Secondary Synchronization Signal (NSSS). Two more channels are offered during the uplink: Narrowband Physical Uplink Shared Channel (NPUSCH), and Narrowband Physical Random-Access Channel (NPRACH) [7,15,16]. NB-IoT’s physical channels, therefore, vary slightly from those of LTE, because NB-IoT’s physical channels and signals are multiplexed over time. Of all these downlink and uplink channels, NB-IoT most benefits the data re-transfer mechanism. This leads to lower-order modulation and the diversity of time to upgrade both the efficiency of demodulation and coverage [17,18]. In each physical subchannel, the Signal to Interference plus Noise Ratio (SINR) for the received signal is computed by considering the noise, transmit power, path loss, and interference in terms of cell scenarios (i.e., microcell, macrocell of a rural area, and macrocell of an urban area) [19,20]. The system assumes Base Stations (BSs) are in vertices at the centre of cells, and that *N* nodes are distributed randomly in the cells.

The Media Access Control (MAC) and networking layers are implemented in conjunction with the LEACH protocol to reduce queuing delays, minimize power consumption, and increase the battery life of devices (i.e., enhance the State of Health (SOH) of the device’s battery). Furthermore, it prevents possible selective forwarding attacks, insider attacks, and node compromise [21,22,23]. The LEACH protocol organizes the networking nodes into clusters and elects some of them as Cluster Heads (CHs). Non-Cluster Head nodes (NCHs) transmit the collected data to the CH using D2D communications. Then, each CH aggregates and prepares the data and sends all to the BS. LEACH initially uses a random selection process for the embedded devices that are chosen to be CHs. This distributes power consumption evenly in the wireless network [21,24]. Intra- and inter-cluster collisions are decreased by the MAC layer, depending on the transmission channel. Orthogonal Frequency Division Multiple Access (OFDMA) is used for uplinking [25] with Binary or Quadrature Phase Shift Keying (BPSK or QPSK) modulation, and Single Carrier Frequency Division Multiple Access (SC-FDMA) is employed for downlinking with QPSK data change [17,26]. Continued monitoring of nodes is necessary when data are initially centralized at the CHs. This makes the D2D clustering method the optimal option. The Key Performance Indicators (KPIs) for this analytical study are the lifetime, throughput, stability period, instability period, abilities sensing range, computation power, and field distribution of a heterogeneous network where the sensor nodes have different energy levels [21]. NB-IoT uses a bandwidth of 180 to 200 kHz [27]. This guarantees efficient communication, and it classifies the data transmitted by the CH into a set of coverage classes. This set connects to a number of signal repeats, which are allocated to users based on path loss during their contact with the BSs [15].

The rest of this paper is arranged as follows: Section 2 gives an overview of D2D communication in cellular networks. Section 3 describes the NB-IoT D2D simulation, modeling, and the node distribution, including the mathematical propagation models, the coverage class definition based on the path loss, the LEACH protocol, the main purpose of the clustering protocol, a few preliminaries on the uplink/downlink queuing server, the latency, the energy consumption model with power profiles for user and BS, and the overall system performance. Section 4 shows the simulation outputs and discusses the obtained results. Finally, we offer concluding remarks and discuss future works in Section 5.

## 2. Overview of D2D Communications in Cellular Networks

A communications device can be used to transmit information, instruct, and establish a connection between a sending and a receiving device. Similarly, Device-to-Device (D2D) communication pertains to a radio technology that allows direct wireless links between mobile users to establish a connection to communicate with each other directly without routing data paths through a network infrastructure. In other words, this type of communication can be used between cellular devices and assists to transmit signals and communicate without traversing the BS. According to study [28], D2D transmission gives a procedure for sending the narrowband user equipment procured information to the BS, efficiently utilizing the spatially close mobile device, which considers transferring hubs to help distribute D2D in task rounds.

All communications between devices must be done through the BS in conventional cellular networks regardless of whether D2D communication is within two mobile users’ reach [29]. Communications between devices through BS sets off customary low data mobile services. For example, these services can include voice calls and texting. Nonetheless, at present mobile users utilize high data mobile rate services for communication. For example, this can contain social media, localizing (position-based services), and live videos, which they might be in a reach for direct communications (short distances) such as D2D communication. Moreover, D2D communications in such situations can enormously expand the network spectral utilization and effectiveness, which aids cellular network performance. Consequently, D2D communications can also enhance energy efficiency, throughput, fairness, higher data rates and latency reduction, as well as offer novel applications [29,30].

In 2012, after the Long-Term Evolution (LTE) Release 12, D2D communication in cellular networks was first introduced to enable peer-to-peer communications between spatially adjacent mobile devices [28]. D2D interaction is normalized in the 3GPP to find and impart between two gadgets. The design allowed a D2D device to discover close-by gadgets. Moreover, LTE also sends information to the anchor BS utilizing resources of the close-by device given by the BS as an alternative once the immediate connection to the BS is impossible. In addition to this, this helps to use re-usability of spectrum effectively. Definitively, this will also play a significant role in accomplishing data sending/receiving with lower power consumption [28]. D2D communication is applicable to IoT, mainly when the power budget of embedded devices prohibits direct communications with the cellular network infrastructure [31].

IoT is an emerging paradigm and refers to a system of interrelated computing devices that can collect and exchange data using embedded sensors over a wireless network without human intervention. In contrast, D2D communication is considered a promising technology to make ultra-low latency communication possible. The concatenation of D2D with the IoT can produce energy-efficient and low-latency interconnected wireless networks. For example, the Internet of Vehicles (IoV) is a D2D-based IoT network where real-time communication occurs among two or more devices. These devices can be cars, smartphones, wearables, or roadside units, among others. Furthermore, the IoV ensures efficiency and safety aspects. For instance, at high speed, a vehicle cautions compact vehicles by D2D communication before the vehicle speed declines or moves to another lane [32]. These little gadgets ought to work over a reasonable distance without devouring an excessive amount of power. These small gadgets exchange small amounts of data/information but consume significant power for communications, compared to data gathering and processing. Therefore, a D2D NB-IoT communication framework seems a viable solution to the problem [31].

According to Al-Samman et al. [33], the expanding number of associated devices is growing significantly and the cellular network structure requires substantial augmentation to absorb that growth. This should be possible by enhancing energy efficiency and proposing a useful and practical framework to use bandwidth and spectrum. Consequently, improved versatility choices for taking care of the expanding number of linked devices ought to be considered. A useful framework is thus required to accomplish this idea to cater for the number of connected devices effectively. The framework should be able to deal with a significant amount of data. Moreover, the whole framework capability should be enhanced w.r.t. current capabilities, by a factor of 100 approximately [33]. Here, D2D communications is an important enabler that is recognized as a paramount technology in developing a beneficial solution for the upcoming sixth-generation wireless network.

## 3. NB-IoT D2D Framework, Model, and Simulation

In this section, detailed information concerning the principles of the NB-IoT D2D framework and simulator are provided. Note that the MATLAB-based NB-IoT simulation framework (i.e., source codes) and its results are publicly available on the MDPI website as supplementary material and on GitHub (https://github.com/Ohood-Althobaiti/NB-IoTD2DSimulation-AnOpen-SourcedFramework, accessed on 4 March 2021).

### 3.1. NB-IoT D2D Architecture

**Figure 1:**
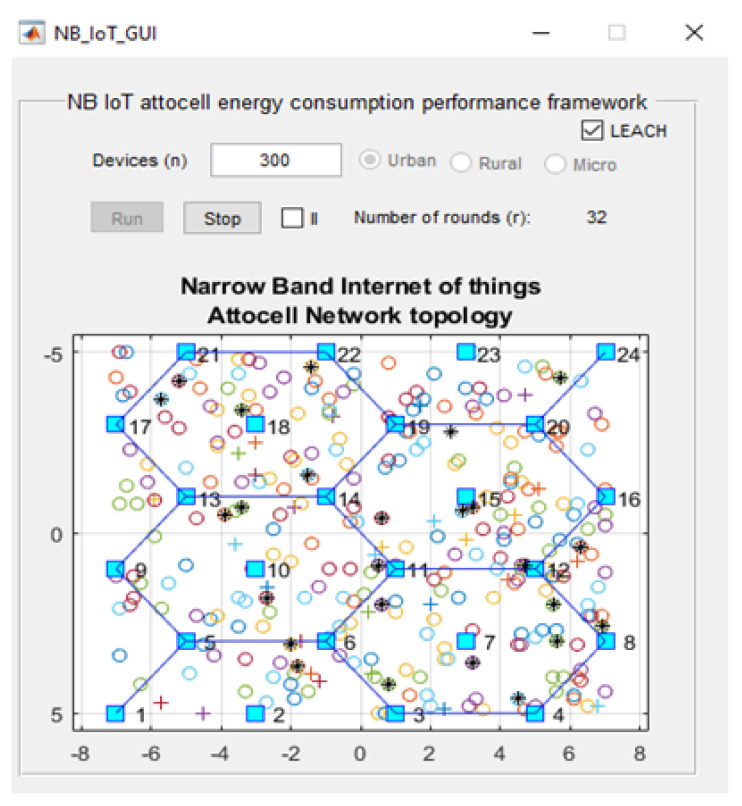
Graphical User Interface (GUI) of the NB-IoT open-sourced simulator.

**Figure 2:**
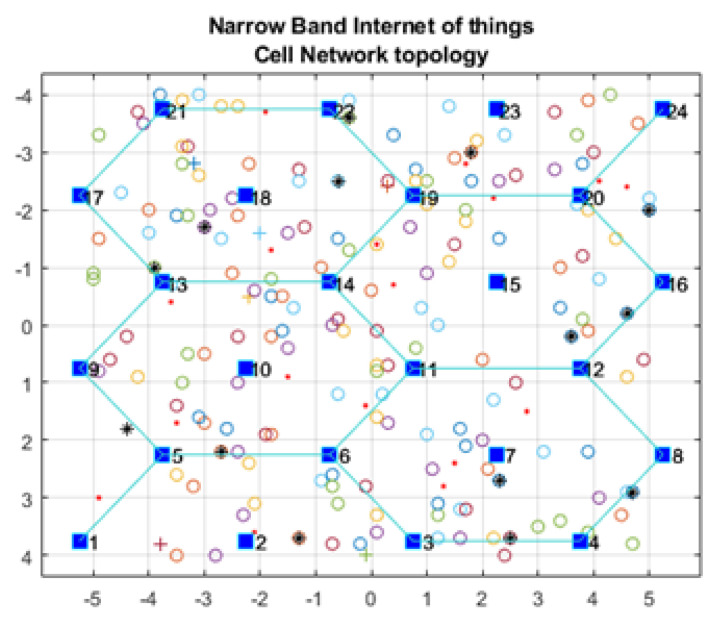
NB-IoT attocell networking topology and field distribution.

**Figure 3:**
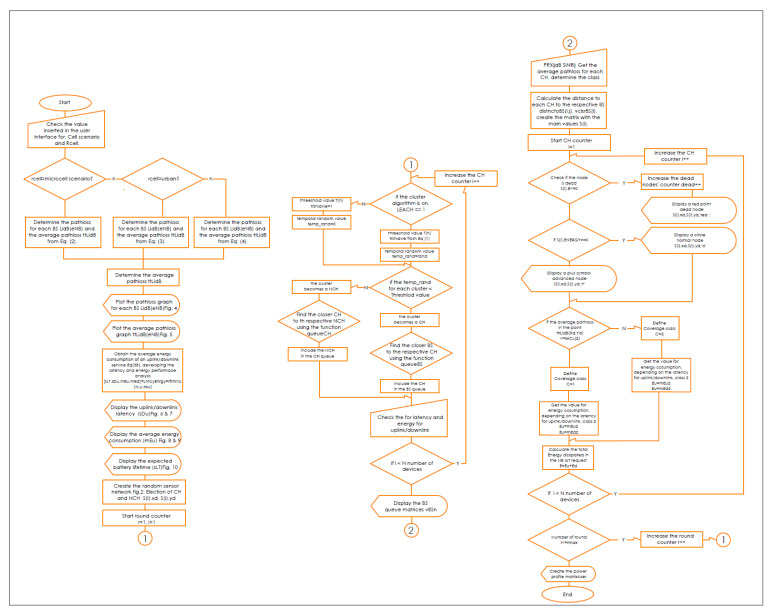
Flowchart of the MATLAB-based NB-IoT D2D simulation.

Our framework was implemented using the Matrix Laboratory Programming Language (MATLAB) [34]. Figure 1 shows the GUI for our NB-IoT attocell networking simulator in the context of three significant cell scenarios: Microcell, macrocell of a rural area, and macrocell of an urban area. The NB-IoT D2D attocell network topology is composed of a grid with 24 BSs that form five cells covering a total area of 80 km^2^. A BS is identified by a blue square. The cell range ($R_{cell}$) is 2 km. The cell is a hexagon bounded by BSs with another BS at the centre. The number of devices/sensor nodes in the field is *n*, and these are placed uniformly. The number of sensor nodes (*n*) can be changed as needed using the GUI. A normal node is represented by a circle (o), and an advanced node resuming the role of a CH is represented by a plus symbol (+). The energy of a normal node is Eo, and the energy of an advanced node equals (1 + α) ×Eo. In this implementation, we assume α equals 1, i.e., the advanced node has double energy to start with. The transmitted packet is referred to by an asterisk (*), a dead node (i.e., a sensor device with a depleted battery) is denoted with a red dot (.), and a half-depleted node is denoted by a red dot in a red circle, as depicted in Figure 2. The flowchart of the proposed MATLAB-based NB-IoT D2D simulation, which is an open-sourced framework, is depicted in Figure 3. Table 1 describes key units in the MATLAB-based NB-IoT D2D simulation framework, and Table 2 summarizes the simulation parameters.

### 3.2. LEACH Routing Protocol

In our simulation, the main purpose of using a single-hop clustering protocol was to downgrade the several-server queuing, decrease inter-cluster collisions, and prevent attacks from malicious nodes. The LEACH protocol is adapted to the NB-IoT D2D setting so as to route network traffic. An upside of using LEACH is that, compared to other traditional multi-hop routing protocols, LEACH is difficult to compromise [21].

Indeed, the nodes around the BS in traditional multi-hop routing procedures are more often engaged, increasing the likelihood of compromise, while only the CHs communicate directly with the BS in LEACH. These CHs are determined randomly in the network, independent of the BS; they also change periodically. As a result, it is very difficult for an attacker to spot the CHs in a LEACH protocol. However, the CHs are responsible for collecting and routing essential data, so a compromised CH is dangerous. An important method for enhancing cybersecurity is key management. While many key distribution systems are available, most are not appropriate for the IoT [35]. For instance, a significant amount of computation is needed for public key-based distribution, a significant amount of storage is required for full pairwise keying, and worldwide keying is threatened. The IoT has limited resources, processing power, communication abilities, and memory, making it difficult to implement cybersecurity mechanisms efficiently.

Returning to the communications principles of LEACH, the protocol selects randomly a few nodes that act as CHs based on a higher energy principle, while the other nodes act as Non-CHs (NCHs). NCHs aggregate data from one or several measurement sensors, and send it to the CH. The aggregated and compressed data are then sent by the CHs to the respective BSs. In this simulation, OFDMA with BPSK or QPSK and SC-FDMA with QPSK are applied for uplink and downlink, respectively, instead of Time Division Multiple Access (TDMA)/Code-Division Multiple Access (CDMA), as used previously in studies involving LEACH.

LEACH is used in the clustering concept to minimize energy dissipation and maximize network lifetime. Consequently, using the LEACH protocol, operations are classified into two stages: Setup and steady steps [36]. The clusters are created in the setup stage, and a CH is elected for each cluster with a probability *p*. The LEACH threshold value T(n) is commonly described by (1). Electing a CH that belongs to the set of embedded devices *G* that were not selected as CH in the last 1/p rounds is achieved based on T(n). Each iteration for electing a CH is called a round, denoted by *r*:

(1) $$T\left(n\right)=\frac{p}{1-p\times (r\times mod(1/p))}\;\forall n\;\in G.$$

Each device selects a random number X∈]0,1[. If X<T(n), then, for the current *r*, this device becomes CH. CHs subsequently broadcast a message to NCHs to request them to connect to their clusters. The NCH devices decide which CH to connect to based on the received signal power, and the NCH transmits an acceptance message to the CHs that will be under its cluster. This communications could also be assisted by the BS but will not change the overall performance and behavior. Thus, each CH establishes an OFDMA/SC-OFDMA schedule to organize transmitting slots between NCH devices in its own cluster based upon certain criteria, such as the number of NCH devices and the collected data type. The schedule of OFDMA/SC-OFDMA is transmitted to all the devices belonging to the cluster.

In the steady stage, the NCH senses data and transmits it to its CH based on the OFDMA/SC-OFDMA schedule. Consequently, the CHs send the sense data to the closest BS. The NB-IoT returns to the initialization (setup) process after a predetermined time, and a new CH is picked.

### 3.3. Channel Propagation Models

The most important IoT signal quality parameters are SINR, Signal-to-Noise Ratio (SNR), and path loss. Thus, we studied the SINR of NB-IoT, demonstrating its relatively high interference immunity compared to e.g. Sigfox and LoRa. Additionally, we simulated the path loss model of NB-IoT in terms of a microcell, a macrocell of an urban area, and a macrocell of a rural area. This NB-IoT D2D framework involves two types of path loss models. First, the path loss between CH and BS (i.e., long range transmission), which was simulated based on [17,19,20,37,38]. Second, the path loss between NB-IoT D2D nodes (i.e., short range transmission between the CH and the respective associated cluster nodes), that was simulated based on [24,31,33,39,40]. The MAC layer verifies whether frames have been received correctly. The communication data link between CHs and BSs is attenuated following the used path loss / propagation models.

**Figure 4:**
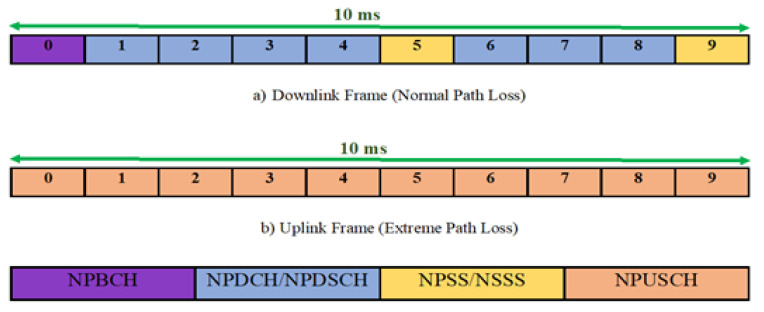
Frame structure of (**a**) NB-IoT downlink OFOMA and (**b**) NB-IoT uplink SC-FDMA.

Figure 4 depicts (a) an NB-IoT downlink OFOMA frame structure and (b) an NB-IoT uplink SC-FDMA frame structure. However, the procedure is decided based on the cell scenario of the intended application. For instance, the path loss pattern of a microcell scenario between CHs and BSs for smart homes, smart devices, and industrial applications is shown in (2) while between NB-IoT D2D nodes is presented in (3):

(2) $${PL}_{CH-BS}=24+45{\;log}_{10}\;\left(d_{eNB}+16\right)$$

(3) $${PL}_{D2D}=\;G_{Rx}+G_{Tx}+{PTX}_{j}+\;\left[4\;\times \;10{\;log}_{10}\left(\frac{6}{4\times \pi \times d_{CH-N}\;}\right)\;\right].$$

Equation (4) illustrates the path loss pattern of an urban macrocell between CHs and BSs for smart cities and suburban areas with smart grids, whereas (5) shows the path loss pattern of an urban macrocell between the CH and the respective associated cluster nodes:

(4) $${PL}_{CH-BS}=103.8+37.6{\;log}_{10}\left(d_{eNB}\right)$$

(5) $${PL}_{D2D}=\;G_{Rx}+G_{Tx}+{PTX}_{j}+\;2.7\;\times \;10{\;log}_{10}\left(\frac{3.5}{4\times \pi \times d_{CH-N}\;}\right).\;\;\;\;\;\;$$

The path loss formula of the rural macrocell, between CHs and BSs, which can be used for smart-farms and wind turbine parks, is demonstrated through (6). And, the path loss formula of the rural macrocell, between NB-IoT D2D nodes is given through (7):

(6) $${PL}_{CH-BS}=96+34.1{\;log}_{10}\left(d_{eNB}\right)\;\;\;$$

(7) $${PL}_{D2D}=\;G_{Rx}+G_{Tx}+{PTX}_{j}+\;2\times \;10{\;log}_{10}\left(\frac{2}{4\times \pi \times d_{CH-N}\;}\right).\;\;\;\;\;\;$$

**Figure 5:**
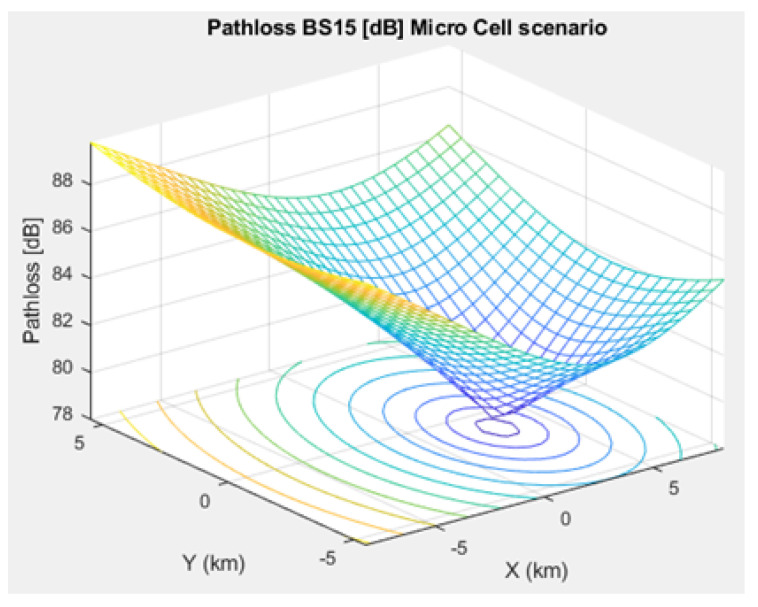
Path loss model for BS No. 15 in a microcell scenario.

**Figure 6:**
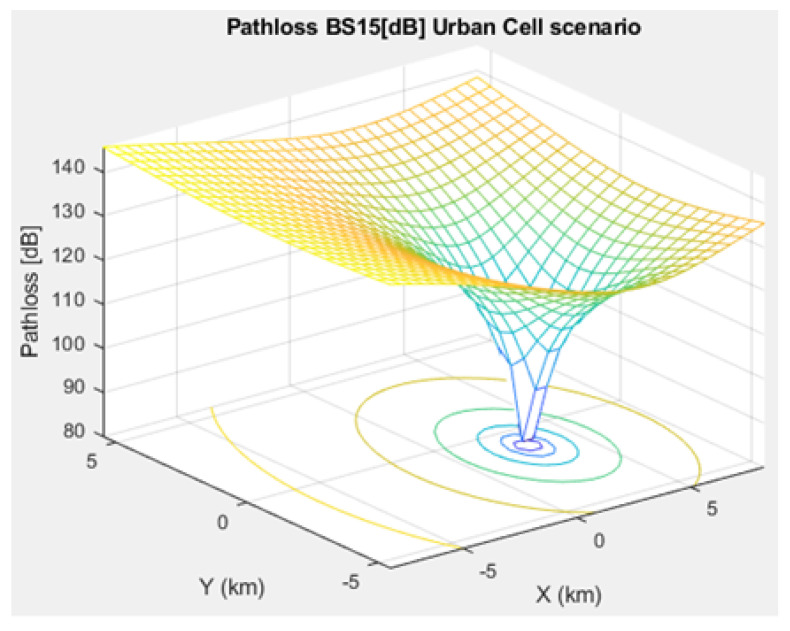
Path loss model for BS No. 15 in an urban macrocell scenario.

**Figure 7:**
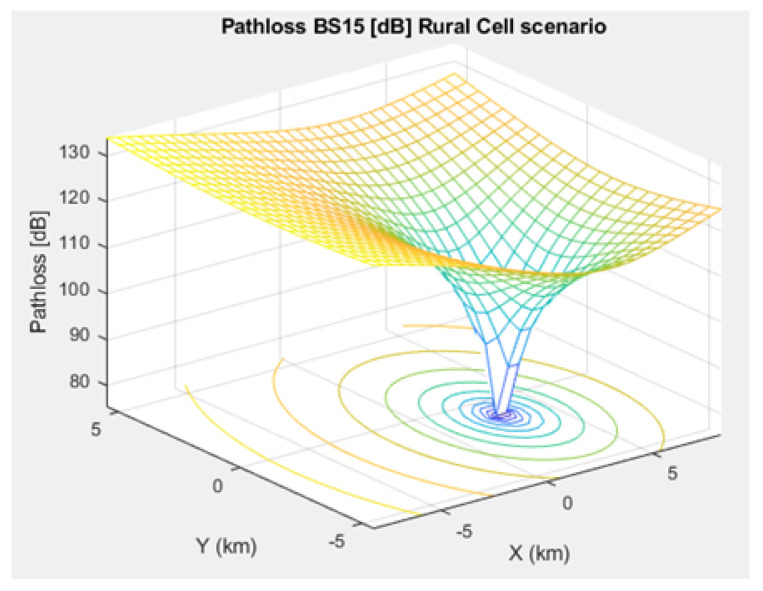
Path loss model for BS No. 15 in a rural macrocell scenario.

Figure 5, Figure 6, and Figure 7 depict the path loss model for a given BS in the microcell, urban macrocell, and rural macrocell scenarios, respectively. The MAC layer reflects the technical features of the channel associated with NCH and CH or CH and BS. In the NB-IoT attocell network simulation, the Maximum Coupling Loss (MCL) of the three coverage zones is 144, 155, and 164 dB. The uplink coverage class uses SC-FDMA with a subcarrier spacing of 3.75 or 15 kHz, while the downlink coverage class applies OFDMA with a subcarrier spacing limited to 15 kHz. Moreover, the data transmission rate for uplink coverage classes ranges between 160 and 200 kbits/s, whereas the data transmission rate for downlink coverage classes ranges between 160 and 250 kbits/s [17]. The effective bandwidth was calculated, and the frequency factor *f* was considered for the purpose of enhancing the SNR as follows [17]:

(8) $$BW\;=\;\frac{180,000\;\mathrm{Hz}}{f},\;\mathrm{where}\;f\;=\;\frac{12}{Tones}$$

(9) $$NsSpctrldnsty\;=\;Nsfigr\;\times \;N_{0}$$

(10) $$\begin{array}{cc} TxSpctrlDnsty\; & =\;{PRX}_{i,j}\;\times \;{BW}^{-1},\mathrm{where}\\ \; & Reception\;power\;of\;equipment\;i\;for\;subchannel\;j\;\\ & denoted\;by\;{PRX}_{i,j}\;=\;\left|{PTX}_{j}\;\times d_{eNB}-\right.\;\left.L-TidB\right|\;\; \end{array}$$

(11) $$SNR\;=TxSpctrlDnsty\times {NsSpctrldnsty}^{-1}.$$

**Figure 8:**
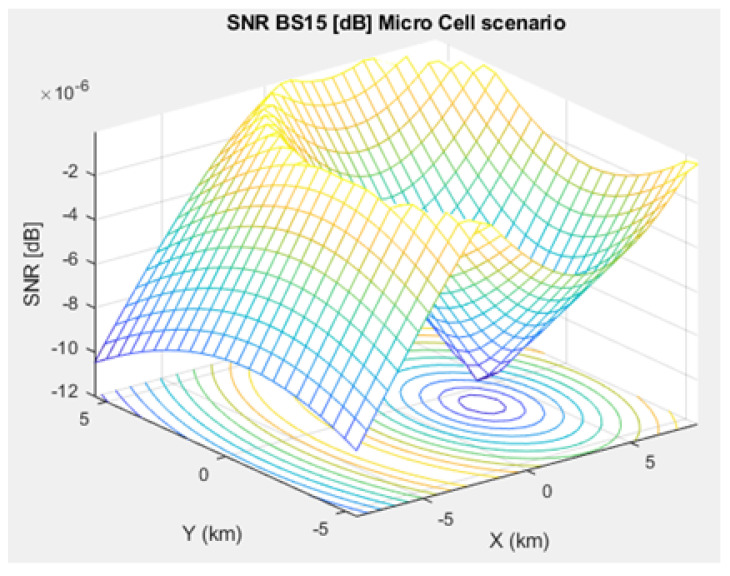
Signal-to-Noise Ratio (SNR) for BS No. 15 in a microcell scenario.

**Figure 9:**
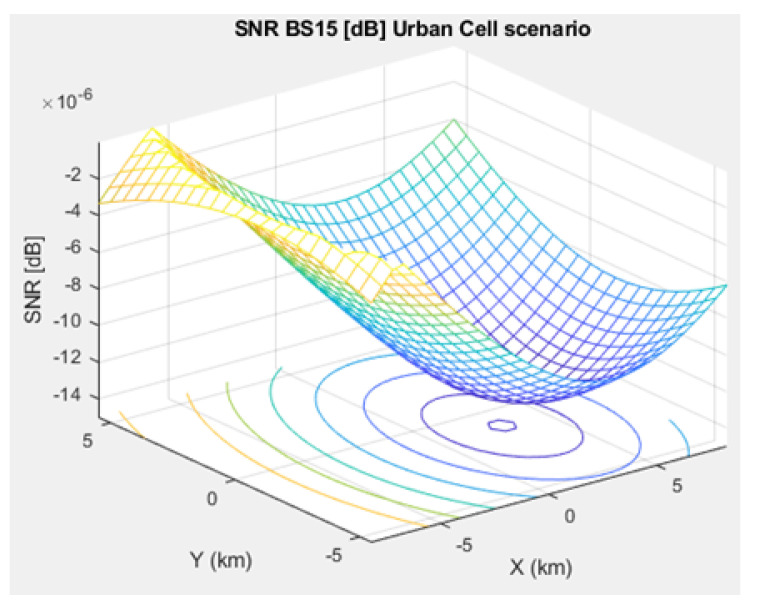
SNR for BS No. 15 in urban macrocell scenario.

**Figure 10:**
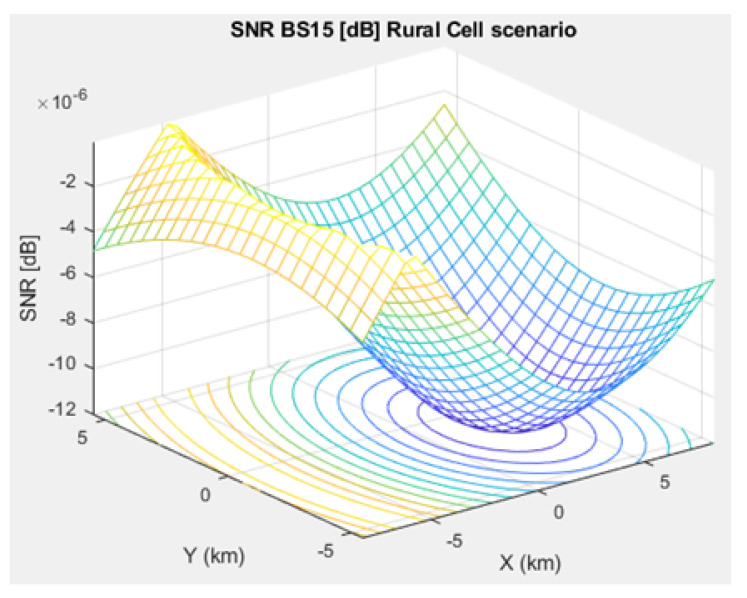
SNR for BS No. 15 in a rural macrocell scenario.

**Figure 11:**
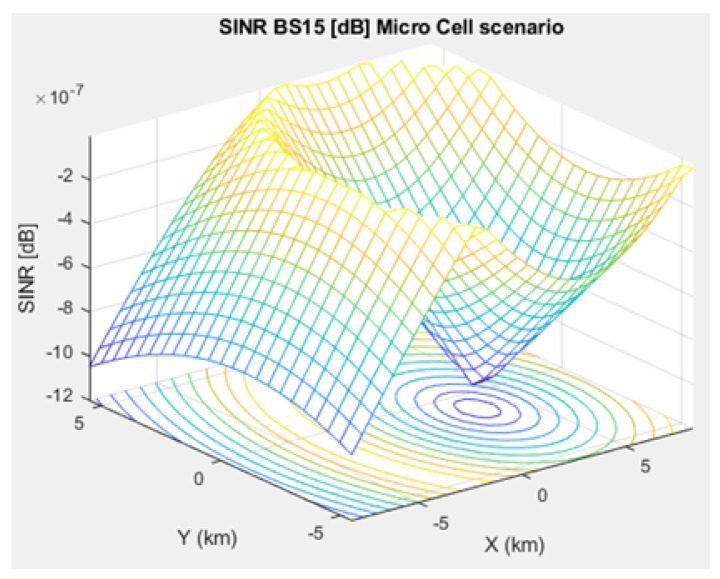
Signal Interference to Noise Ratio for BS No. 15 in a microcell scenario.

**Figure 12:**
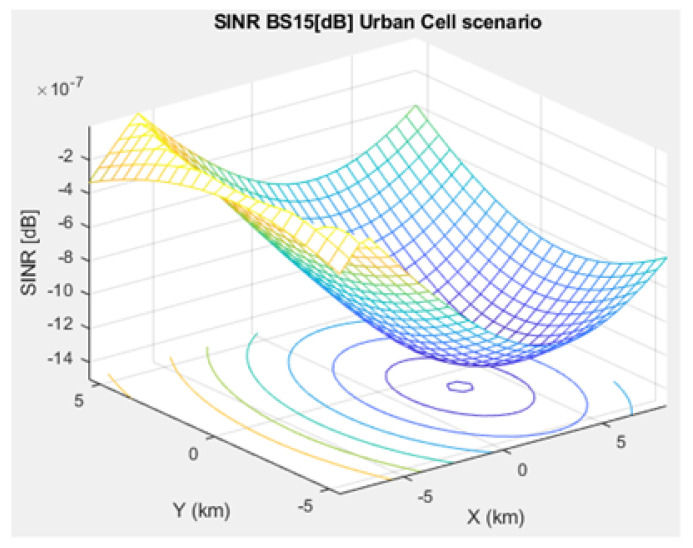
Signal Interference to Noise Ratio for BS No. 15 in an urban macrocell scenario.

**Figure 13:**
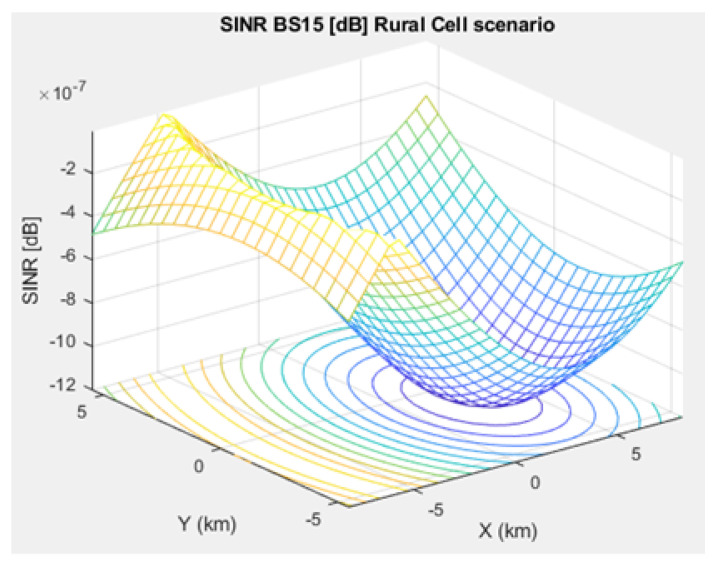
Signal Interference to Noise Ratio for BS No. 15 in a rural macrocell scenario.

The SNR for a given BS in the microcell, urban macrocell, and rural macrocell scenarios is presented in Figure 8, Figure 9, and Figure 10, respectively. In view of the communication channel capacity principle, the data transmission rate is measured by the SINR for the signal. When the CH receives or sends frames, the SINR is calculated as per (12) [19]. Figure 11, Figure 12, and Figure 13 illustrate the SINR for the a given BS in the microcell, urban macrocell, and rural macrocell scenarios, respectively.

(12) $$SINR_{i,j}=\frac{PRX_{i,j}}{(NsSpctrldnsty\times B)+I}.$$

### 3.4. Latency-Energy Model

**Figure 14:**
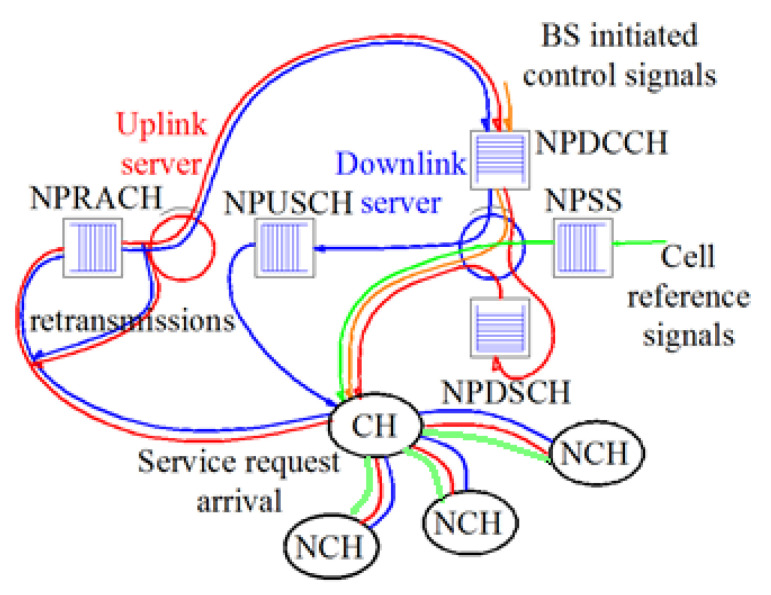
LEACH-based NB-IoT traffic queuing model.

NB-IoT can handle three coverage classes, i.e., robust (extended), extreme, and normal, for serving devices with constrained resources and suffering varying path loss levels [41]. However, the requirements of latency and throughput are maintained in the extreme coverage class, whereas improved performance is ensured in the extended or normal coverage class [42]. Therefore, in this work, we focus on normal and extreme coverage classes. This latency-energy model is taken from [15,43]. A total of two coverage classes are defined as C=2. On the basis of estimated path loss, a class is assigned to a device by the BS that informs the assigned device of the dedicated path between them. Class *j* and ∀j are supported by the replicas number cj, which are transmitted based on data and the control packet [15]. The uplink/downlink access technique is modeled as two servers working for the associated traffic queues, as shown in Figure 14 [15]. For the purpose of brevity, interested readers are referred to [15] for further information on Figure 14. The reserved NPRACH period of class *j* is denoted by cjτ. The unit length τ of the NPRACH for the class of coverage is denoted by cj=1. The average time duration between the NPDCCH occurrences is represented by td in this latency–energy model.

Those signals that are multiplexed with respect to time undergo trade-off analysis in the NP channel using the down and uplink algorithm under the assumption that the coverage classes in the network are the two represented by C=2. The first coverage class corresponds to the CH with the normalized path loss, while the other corresponds to clusters having extreme path loss. The path loss of the second group of clusters is in the communication and in the evaluation of trade-offs. Considering the presence of the available radio resources of the NB-IoT subsystem and the amount of traffic data, the scheduling is optimized with the minimum latency, extended battery lifetime, and reduced power performance necessary to maintain the existing scheduling policy.

The number of communication sessions corresponds to the speed of arrival of the uplink and downlink requests made in a day by the specific device to the given system. The rate depends on the number of sessions that a cluster achieves daily and is denoted of service requests by the following:

(13) $$G_{u}=Sp_{u}N,$$

where *S* is the required sessions per day and $p_{u}$ is the probability of the uplink requests for the service.

For the $G_{d}$, the probability of downlink becomes $1-p_{u}$, which produces the equation as represented in (14):

(14) $$G_{d}=\left(1-p_{u}\right)\;N\;S.$$

Synchronization is required by CH for the uplink and downlink services with the BS on top of NSSS and NPSS, where the synchronization delay required is $D_{syj}$; $D_{sy1}$ = 0.33 s, $D_{sy2}$ = 0.66 s and also the power consumption of $P_{l}=0.1$, to make the listening represented by $E_{syj}$:

(15) $$E_{{sy}_{j}}=D_{{sy}_{j}}P_{l}.$$

A Random-Access request (RA) is sent by a CH, and the BS responds with a Random Access Response (RAR) containing the NPDCCH message. A device connects to the BS in a deep sleep mode and reconnects for the transmission of RA messages along with a random number. The number of attempts made depends on the probability of available resources and is denoted by $N_{max}$, whereas the probability $P_{j}$ depends on the class of the devices that are attempting to connect to the system. $D_{rrj}$ represents the closed formula for latency:

(16) $$E_{ra_{j}}=\left(\;\xi \;P_{t_{j}}+P_{c}\right)c_{j}\tau +P_{I}\left(D_{ra_{j}}-c_{j}\tau \right)$$

(17) $$E_{rar_{j}}=D_{rar_{j}}\;\times \;P_{l}$$

(18) $$D_{rr_{j}}=\sum_{l=1}^{N_{rmax}}P_{j}l{\left(1-P_{j}\right)}^{l-1}\left(D_{rarj}+D_{raj}\right)$$

(19) $$E_{rr_{j}}=\sum_{l=1}^{N_{r_{\max}}}P_{j}{\left(1-P_{j}\right)}^{l-1}\left(E_{rar_{j}}+E_{ra_{j}}\right).$$

The latency expected in the system is denoted by $D_{raj}$ and $D_{rarj}$ and corresponds to the transmitted requests and the RAR, respectively. In light of having cj of the order of repetition in the coverage class of $c_{1}=1$, $c_{2}=2$, the average length (denoted by τ) of the request signaling is 10 ms.

The equations for $D_{raj}$ and $D_{rarj}$ are as follows:

(20) $$D_{{ra}_{j}}=\tau c_{j}+\;\frac{1}{2}t$$

(21) $$D_{{rar}_{j}}=\frac{1}{2}\;td+uc_{j}+\;\frac{1}{2}D_{t}Q.$$

*Q* is the number of requests that the server can handle in the queue. This value determines the average wait time before the incoming RA request is handled and is represented by $D_{w}$:

(22) $$D_{w}=\frac{1}{2}\;Q\;D_{t}$$

(23) $$Q=\sum_{J=1}^{C}\left[f_{j}\left(G_{d}+G_{u}\right)\max\left\{t_{j},td\right\}\right]+\left(td\;\times \;\lambda_{bs}\right).$$

In the downlink channel (NPDCCH), the service time is denoted by $D_{t}$. The time for control frame sending is *u*, and the time for class *j* transmission is $D_{tj}=cj\;\times \;u$:

(24) $$D_{t}=\sum_{j=1}^{C}f_{j}D_{tj}\;;\;D_{t}=\sum_{j=1}^{C}f_{j}uc_{j}.$$

If the class of node is represented by *j* and the orthogonal preambles in every *t* seconds are $M_{j}$ having a content of average $N_{j}$ CH nodes, then the equation corresponds to:

(25) $$N_{j}=f_{j}\left({G_{d}+G}_{u}\right)t_{j}.$$

$P_{jRACH}$ is written as:

(26) $$P_{jRACH}\sum_{K=2}^{N}\frac{{\left(N_{j}\right)}^{k}\;({M_{j}-1)}^{k-1}e^{{-N}_{j}}}{k!\;({M_{j})}^{k-1}}.$$

The service time in the distribution function for the system $\left(F_{1}\left(x\right)\right)$ and the total sum in time for n>1 nodes where the unit step function is represented by H(x):

(27) $${\;F}_{1}\left(x\right)=\sum_{j=1}^{C}H(x-c_{j}u)f_{j}$$

(28) $${\;F}_{n}\left(x\right)=\sum_{j=1}^{C}F_{n-1}\left(x-c_{j}u\right){\;f}_{j}.$$

The message (RAR) received with a probability of $P_{jRAR}$ within $T_{th}$ shows the number of queue requests that potentially need to be served by *K*, as follows:

(29) $$P_{jRAR}=1-\sum_{K=2}^{\infty }\sum_{k=1}^{K-1}\frac{Q^{K}k}{e^{Q}\times K\times K!}\;(F_{K-k-1}\left(T_{th}\right))\;\left(1-F_{K-k}\left(T_{th}\right)\right).$$

The uplink channel (NPUSCH) is the queue system that looks into the requests that are allocated in fraction *w*. The value of *w* can be calculated as follows:

(30) $$w=1-\sum_{j=1}^{C}{\left({\tau \;c}_{j}\right)\times \left(t_{j}\right)}^{-1}.$$

The NPUSCH can be modeled as a Batch Poisson Process (BPP), which results in the modelled arrival of requests for service to the NPUSCH because of the reservation of the NPRACH periods in the whole system.

The mean size of batch G is calculated as follows:

(31) $$G=\frac{\sum_{j=1}^{C}f_{j}t_{j\;}G_{u}}{C}.$$

The time taken by the uplink packet transmission goes along a general distribution, with $S_{a}$ and $S_{b}$ as the first two moments.

(32) $$S_{a}=\frac{1}{w}\sum_{j=1}^{C}\frac{f_{j}l_{a}c_{j}}{R_{j}}$$

(33) $$S_{b}=\sum_{j=1}^{C}\frac{c_{j}^{2}f_{j}l_{a}}{\;{\left(wR_{j\;}\right)}^{2}}.$$

The average transmission rate of the uplink data for class *j* is denoted by Rj. Thus, CHs send and receive the corresponding field data using NPUSCH or NPDSCH according to the following equations:

(34) $$D_{{tx}_{j}=}\frac{R_{j}w\;\rho S_{b}+\;R_{j}w\;G\;{\left(S_{a}\right)}^{2}+\;2S_{a}\left(1-\rho \right)\;c_{j}l_{a}}{2{R_{j}w\;S}_{a}\left(1-\rho \right)}$$

(35) $$\rho =\sum_{j=1}^{C}S_{a}G\times {\left(t_{j}\right)}^{-1}$$

(36) $$E_{{tx}_{j}}=P_{I}\left(\frac{{R_{j}wD}_{txj}-c_{j}l_{a}}{wR_{j}}\right)+\frac{\left(\xi P_{t_{j}}+\;P_{c}\right){\;l_{a}\;c}_{j}}{wR_{j}}.$$

The downlink shared channel (NPDSCH) is modeled as a system for handling the queue wherein the server responds to all of the request messages by visiting them one at a time. The time when the NPBCH, NPDCCH, NSSS, and NPSS are not scheduled is a fraction represented by *y*:

(37) $$y=\frac{1}{td}\left(td-b\times td-Q\right)\sum_{j=1}^{C}c_{j}f_{j}u.$$

The mean size of batch *g* can be modelled as BPPs that are sent for service request by a downlink to the queue of NPDSCH:

(38) $$g=\sum_{j=1}^{C}\frac{f_{j}t_{j}G_{d}}{C}.$$

If a general distribution of $m_{a}$ and $m_{b}$ is followed by the length of the packet, the initial two moments having a distribution of packet duration of transmission are $h_{a}$ and $h_{b}$ as follows:

(39) $$\;\;h_{a}=\frac{1}{{y\;R}_{j}}\sum_{j=1}^{C}m_{a}f_{j}c_{j}\;\;$$

(40) $$h_{b}=\frac{1}{{\left({y\;R}_{j}\right)}^{2}}\sum_{j=1}^{C}{m_{b}f}_{j}c_{j}^{2}.$$

$N_{j}^{2}$ is the average downlink rate of data from class *j*. The latency in received data $D_{{rx}_{j}}$ can be calculated as follows:

(41) $$v=\frac{1}{t_{j}}\sum_{j=1}^{C}h_{a}\times \;G$$

(42) $$D_{{rx}_{j}}=\;\frac{N_{j\;}\;y\;\;v\;\;h_{b}+N_{j}\;\;y\;\;g\;\;{\left(h_{a}\right)}^{2}+\;{2\;\;h_{a}\left(1-v\right)\;\;c}_{j}{\;\;m}_{b}}{2\;\left(1-v\right)\;h_{a}\;N_{j\;}y}.$$

**Figure 15:**
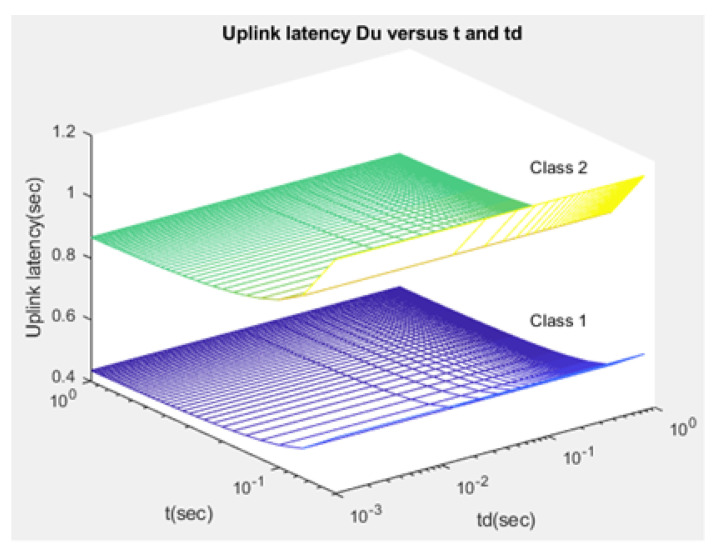
Latency of uplink service request—$D_{uj}$.

**Figure 16:**
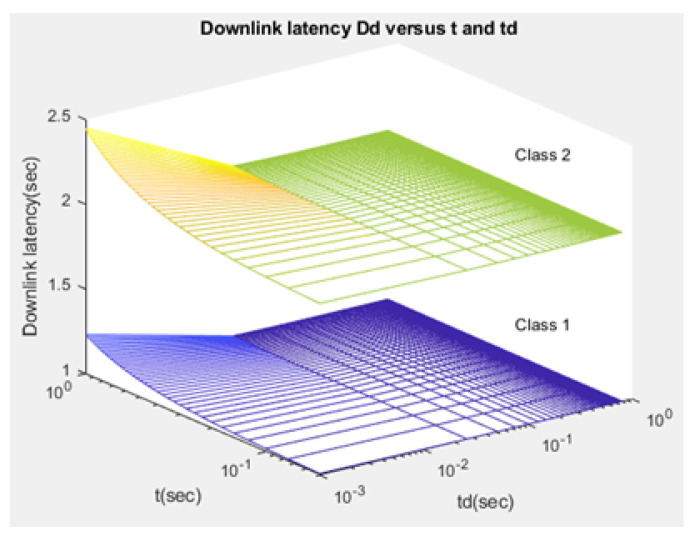
Latency of downlink service request—$D_{dj}$.

The total expected latency $D_{uj}$,$D_{dj}$ for an uplink/downlink service request of class *j* (class 1 for normal path loss and class 2 for extreme path loss) depends on the communication time duration of the consecutive scheduling of NPRACH is *t*. The time duration of two consecutive schedulings of NPDCCH is td. When the CH sends the request, it consumes time while it waits on the queue, as depicted in Figure 15 and Figure 16:

(43) $$D_{uj}=D_{{sy}_{j}}+\;D_{{tx}_{j}}+\;D_{{rr}_{j}}$$

(44) $$D_{dj}=D_{{sy}_{j}}+D_{rxj}+\;\;D_{{rr}_{j}}.$$

**Figure 17:**
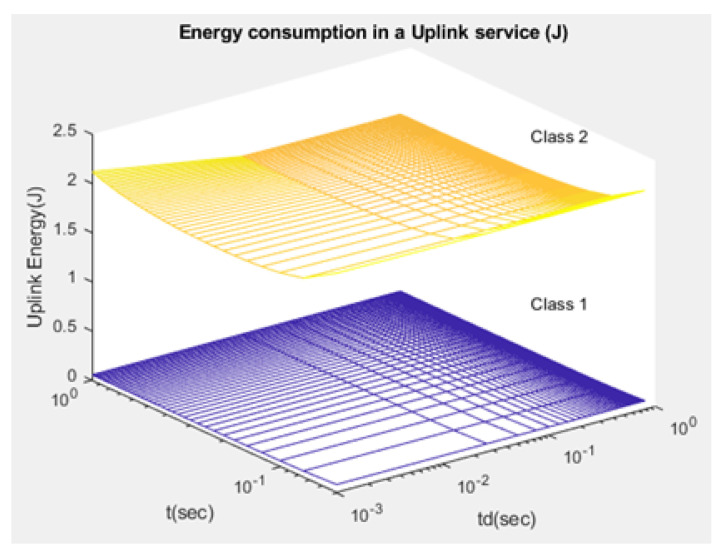
Energy of uplink service request—$\xi_{uj}$.

**Figure 18:**
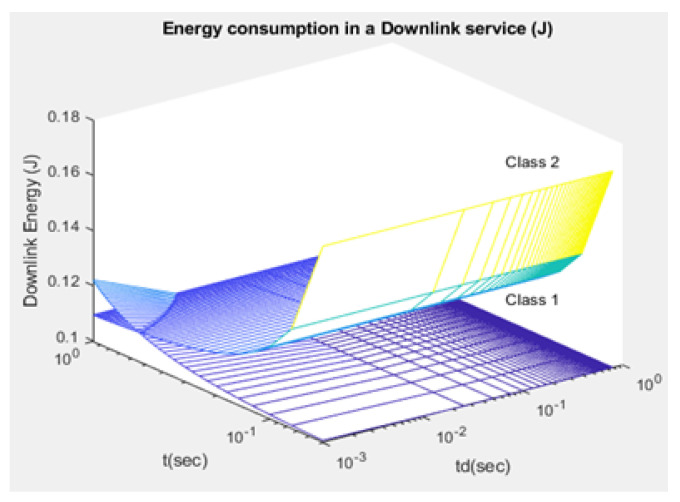
Energy of downlink service request—$\xi_{dj}$.

The average consumption of energy in the uplink and downlink services is denoted by $\xi_{uj}$,$\xi_{dj}$, respectively (Figure 17 and Figure 18):

(45) $$\xi_{uj}=E_{{tx}_{j}}+E_{{rr}_{j}}+E_{{sy}_{j}}$$

(46) $$\xi_{dj}=E_{{rx}_{j}}+E_{{rr}_{j}}+E_{{sy}_{j}}.$$

**Figure 19:**
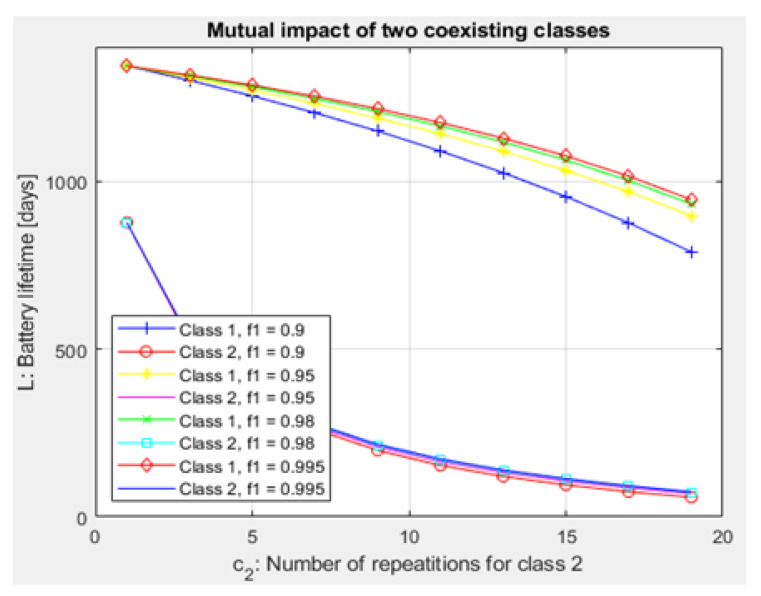
Expected lifetime of devices batteries with respect to the number of repetitions.

**Figure 20:**
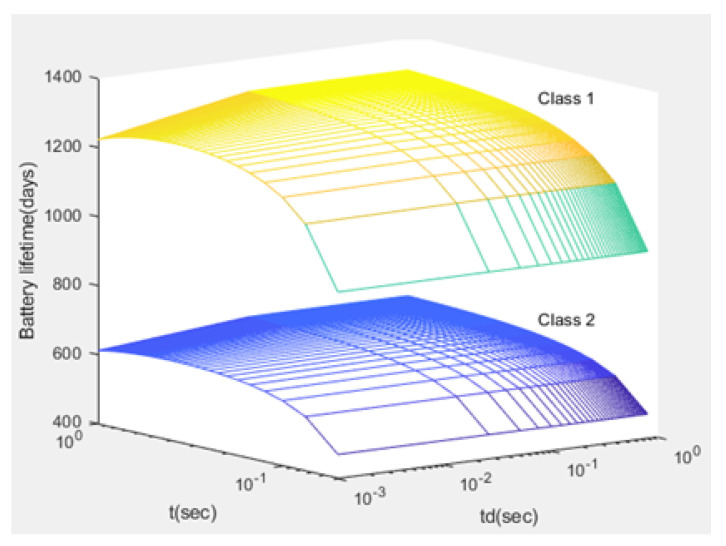
The lifetime of a device’s battery $L_{j}$ versus *t* and td.

Finally, for each CH, the $L_{j}$ (expected lifetime of battery) is formulated using (47) and is displayed in Figure 19 and Figure 20:

(47) $$L_{j}=\frac{E_{o}}{\xi_{uj\;}p_{u}\;S+\left(1-p_{u}\right){S\;\xi }_{dj}}\;\left[days\right].$$

## 4. Simulation Output and Discussions

**Figure 21:**
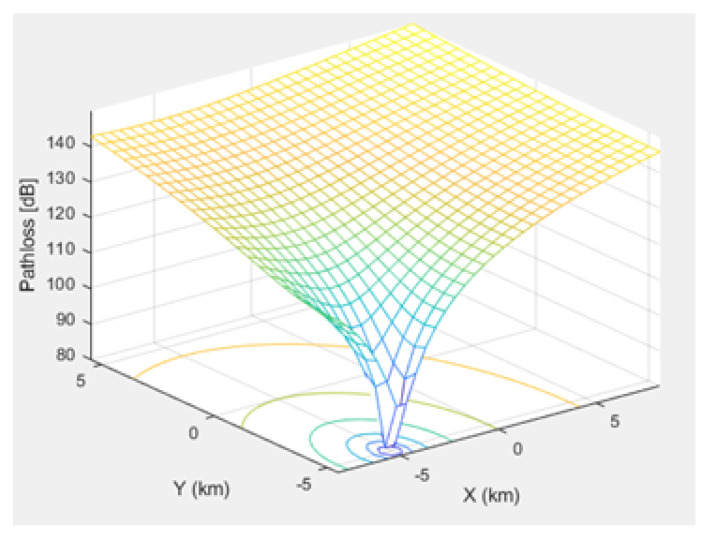
Path loss in the urban scenario, BS No. 21.

**Figure 22:**
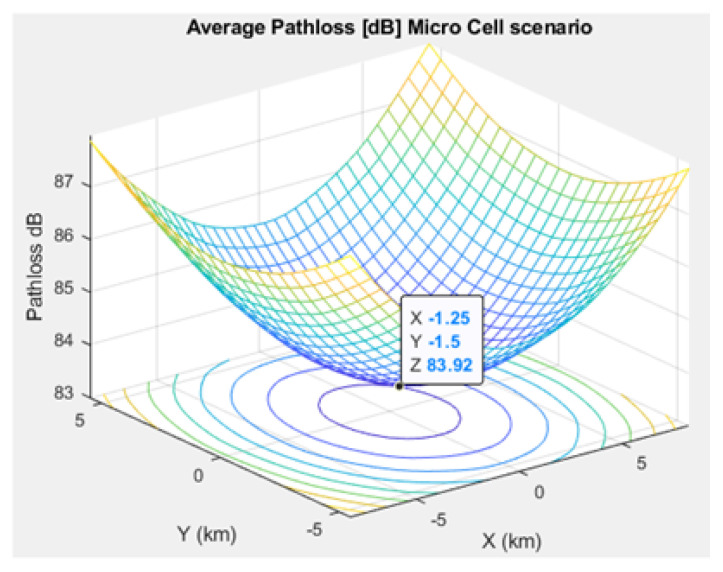
Average path loss in the microcell scenario.

**Figure 23:**
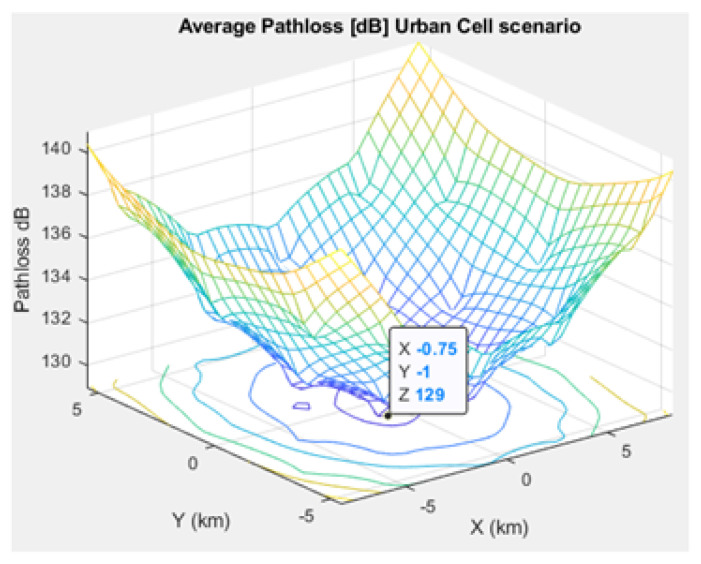
Average path loss in the urban scenario.

**Figure 24:**
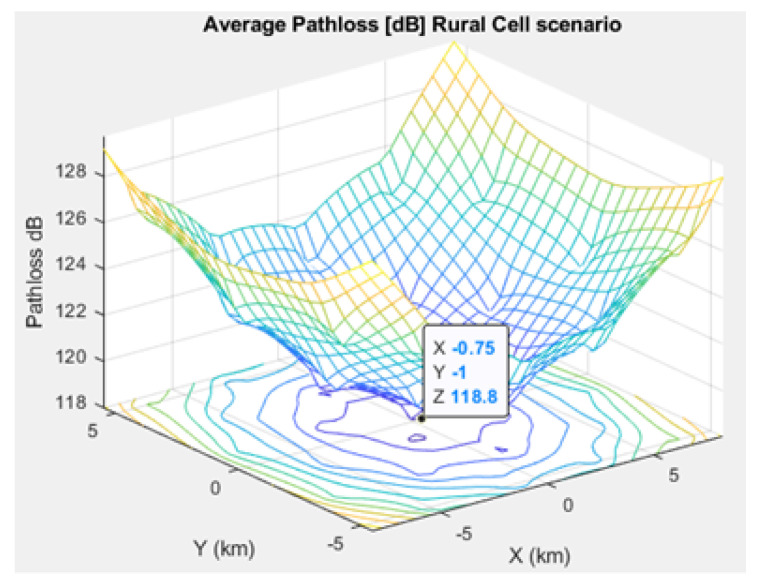
Average path loss in the rural cell scenario.

Compared to studies such as [15,43], the proposed NB-IoT D2D simulation minimizes the latency from uplink/downlink service requests, as presented in Figure 15 and Figure 16. Our framework also reduces the energy consumption that comes from the uplink/downlink service request, as shown in Figure 17 and Figure 18. Furthermore, it improves the coexistence between normal and extreme coverage classes, as illustrated in Figure 19, and enhances the lifetime of the NB-IoT device’s battery, as illustrated in Figure 20. Based on the cell radius and selected propagation scenario, our open-sourced model presents the path loss graph, as illustrated in Figure 21. As a result, the IoT system designer can use our model to develop the most accurate estimations, allowing for a decrease in energy consumption and an increase in performance by varying the simulator parameters. Figure 22, Figure 23 and Figure 24 show the path loss average between CHs and BSs for the NB-IoT D2D simulation framework compared with the MCL.

As shown in Figure 20, the maximum lifetime for class 1 is td=0.526 and t=1.00, whereas the maximum lifetime for class 2 is td=0.626 and t=1.00. Figure 15 illustrates that the minimum latency of an uplink for class 1 is td=0.001 and t=1.00, while the minimum latency of an uplink for class 2 is td=0.001 and t=1.00. As shown in Figure 16, the minimum latency of a downlink for class 1 is td=0.051 and t=0.05 however, the minimum latency of a downlink for class 2 is td=0.051 and t=0.05.

**Figure 25:**
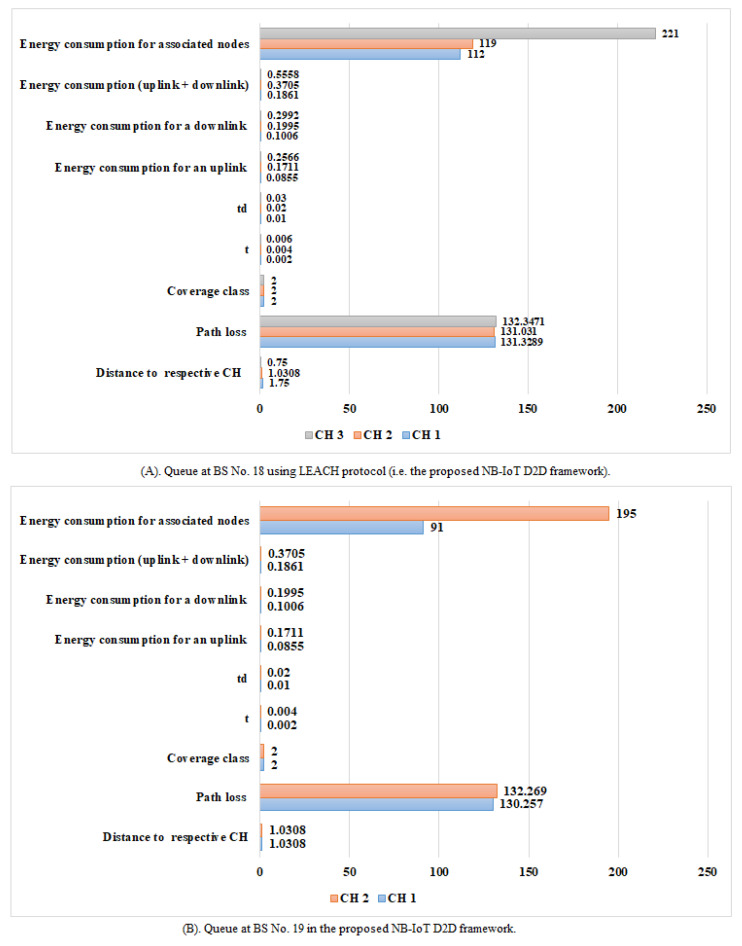
Queue at BSs No. 18 and No. 19 in the proposed NB-IoT D2D framework.

**Figure 26:**
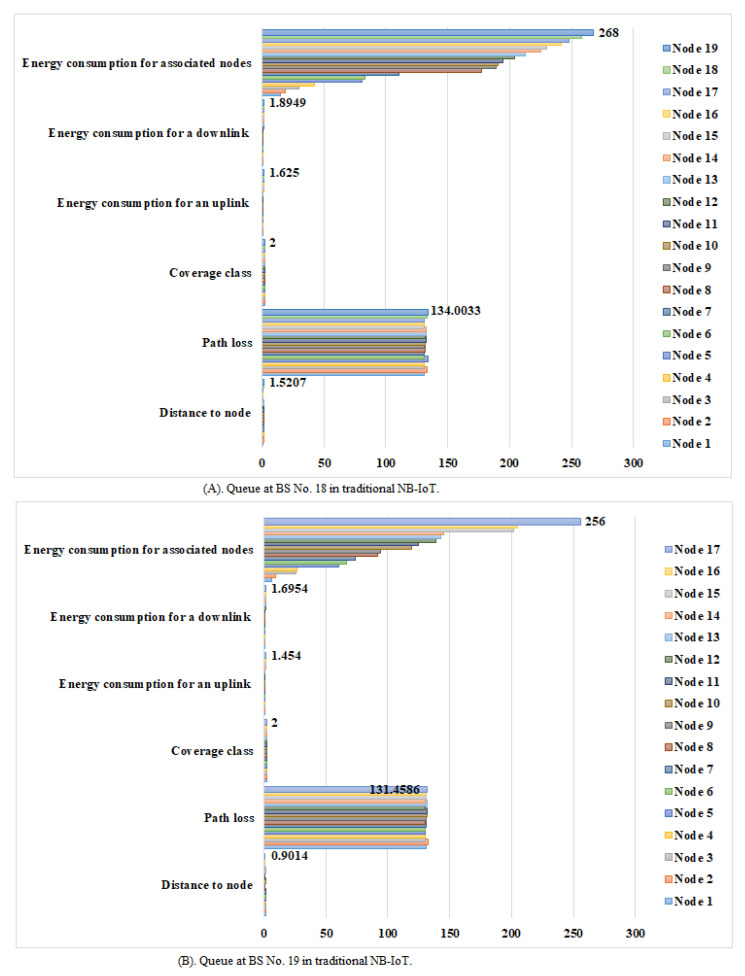
Queue at BSs No. 18 and No. 19 in a traditional NB-IoT.

The NB-IoT attocell network topology integrates with the LEACH algorithm to reduce several queues to a few (Figure 25 vs. Figure 26). This NB-IoT D2D framework, as shown in Figure 25A,B, produces a BS queuing matrix (vBSnx) that shows the total expected energy consumption for the respective associated nodes, the total expected energy consumption for uplink and downlink services, the devices’ energy consumption for a downlink request $\left(\xi_{dj}\right)$, the device’s energy consumption for an uplink request $\left(\xi_{uj}\right)$, the time between two NPDCCH schedules (td), the time between two NPRACH schedules (t), the type of coverage class, the path loss during communication, and the distance to the respective CHs, respectively. Figure 26A,B explains the queue at BS No. 18 and BS No. 19 in case of traditional NB-IoT (i.e., without using LEACH protocol). It shows the total expected energy consumption for the respective associated nodes, the devices’ energy consumption for a downlink request $\left(\xi_{dj}\right)$, the device’s energy consumption for an uplink request $\left(\xi_{uj}\right)$, the type of coverage class, the path loss during communication, and the distance to the node. The queuing matrix (vBSnx) can indicate the queue at each BS in case of the NB-IoT D2D and conventional NB-IoT, but BS No. 18 and BS No. 19 have been randomly selected as examples. Figure 25A confirms there are only three CHs queuing at BS No. 18, and Figure 25B shows there are only two CHs queuing at BS No. 19). Figure 26A,B shows there are 19 nodes queuing at BS No. 18 and 17 nodes queuing at BS No. 19, respectively. This proves practically that when the LEACH model is used, the queues at each BS are minimized. This reduces the latency (i.e., delay time) and energy consumption at each BS.

**Figure 27:**
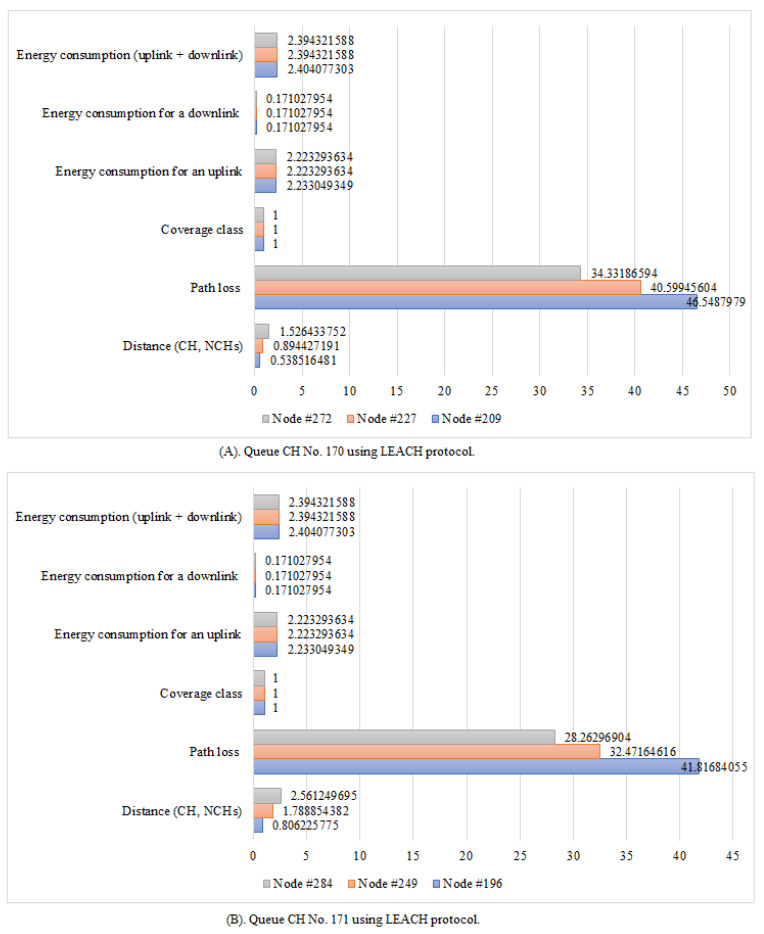
Queue CHs No. 170 and No. 171 using LEACH protocol.

In addition, there is a queue at each CH (mtrxCH(:,:,n)), as illustrated in Figure 27A,B. It displays the total expected energy consumption for uplink and downlink services, the devices’ energy consumption for a downlink request $\left(\xi_{dj}\right)$, the device’s energy consumption for an uplink request $\left(\xi_{uj}\right)$, the coverage class, the path loss between devices, the distance between CH and its NCHs, and the respective associated cluster nodes. The queuing matrix (mtrxCH(:,:,n)) can explain the queue at each CH in the proposed NB-IoT D2D simulator but CH No. 170 and CH No. 171 have been chosen randomly as an example (Figure 27A,B). As a result, the values of parameters such as energy consumption and delay time are reduced because the path loss between users’ equipment is generally applied in class 1.

**Figure 28:**
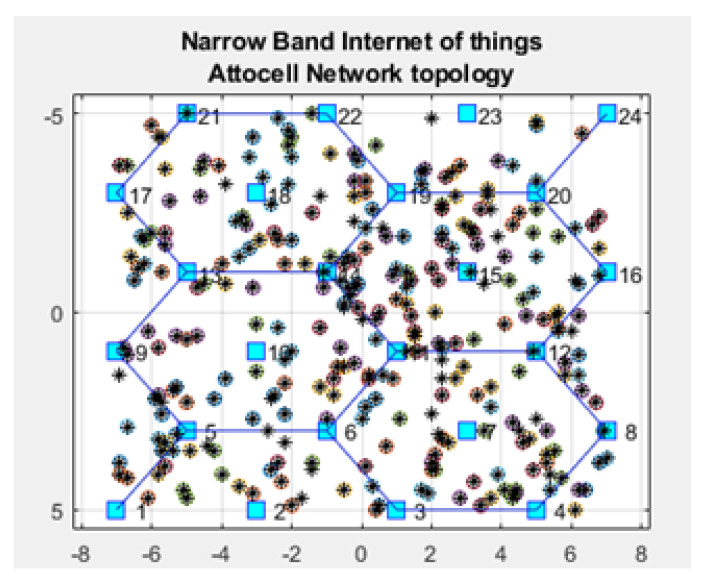
NB-IoT network without a LEACH scheme showing all nodes sending data at the same time (300 users).

**Figure 29:**
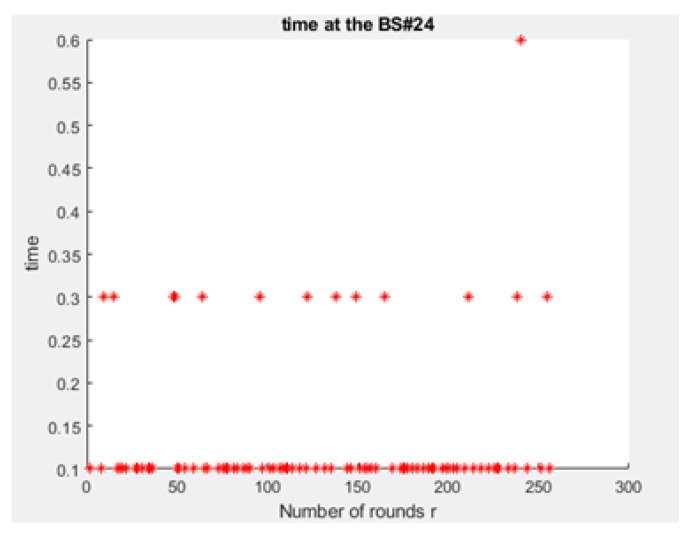
Power profile of advanced node (power consumption for user’s device No. 23).

**Figure 30:**
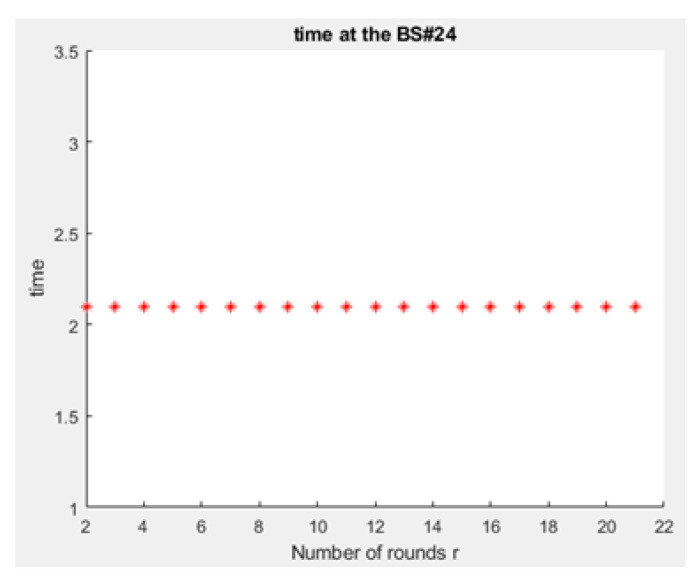
Power profile of a normal node (power consumption for user’s device No. 50).

As demonstrated in Figure 26 and Figure 28, the queues at the BSs are longer when the LEACH routing algorithm in the network layer of NB-IoT is not used because all the devices in the traditional NB-IoT attocell network topology submit uplink/downlink requests at the same time. This causes the energy dissipation in the traditional NB-IoT attocell communications to amplify, and the delay time rises (Figure 29 vs. Figure 30), degrading the battery lifetime. It can be observed that path loss increases proportionally to the distance. Consequently, many nodes in the NB-IoT attocell network topology need to connect using class 2, which also increases the energy consumed. In the LEACH protocol scenario, however, each NCH selects the nearest CH to join to its cluster, and thus most communications are through class 1 (Figure 27).

**Figure 31:**
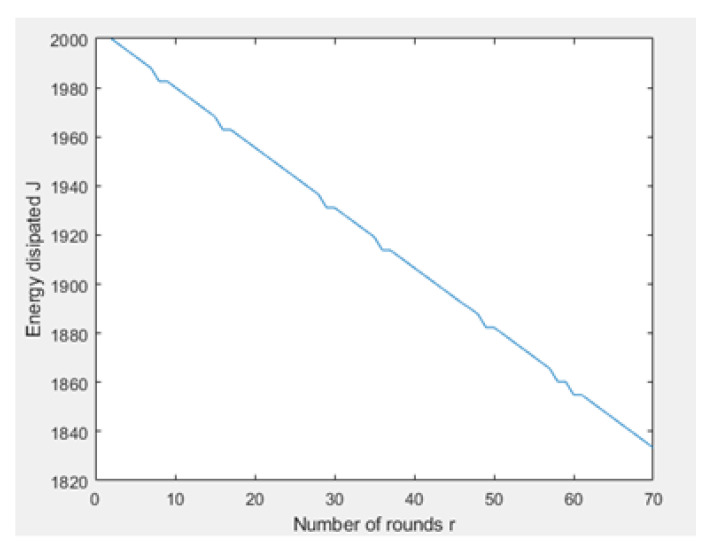
Delay time at BS No. 24 in proposed NB-IoT D2D framework.

**Figure 32:**
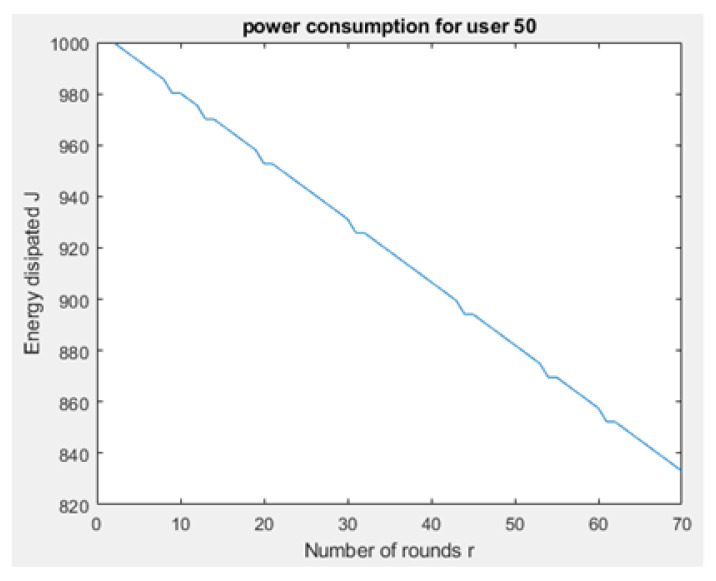
Delay time at BS No. 24 in conventional NB-IoT.

**Figure 33:**
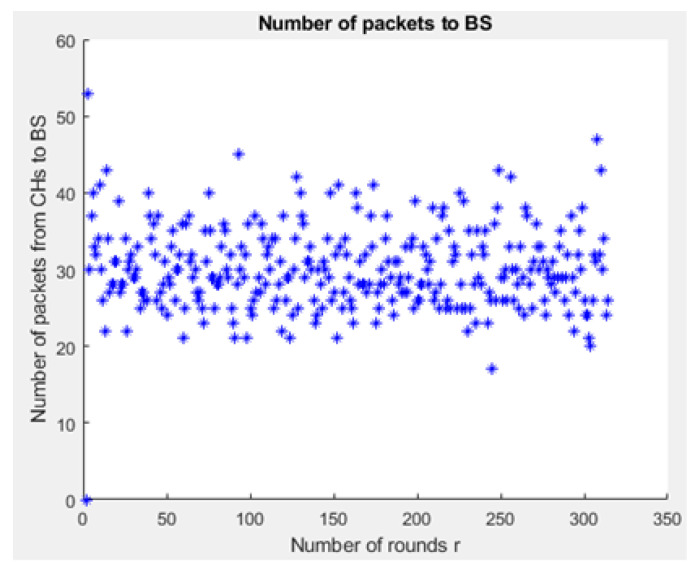
Number of packets per round *r* in NB-IoT D2D attocell network.

**Figure 34:**
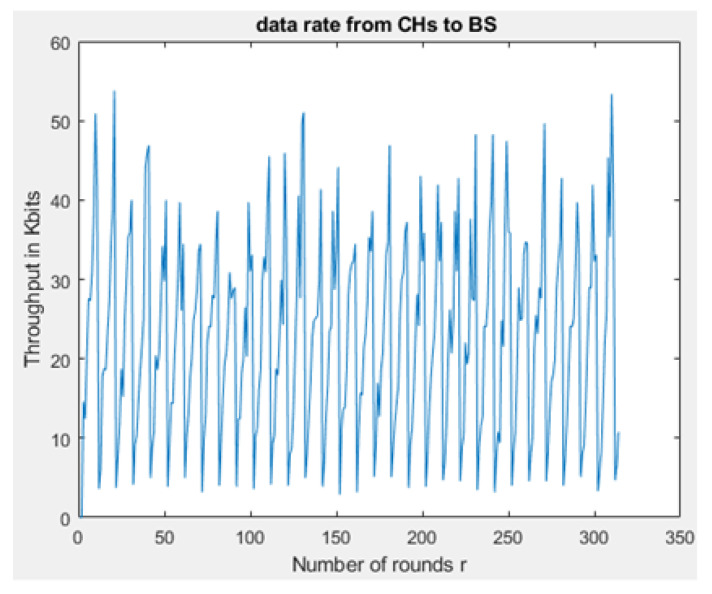
NB-IoT D2D attocell network throughput.

**Figure 35:**
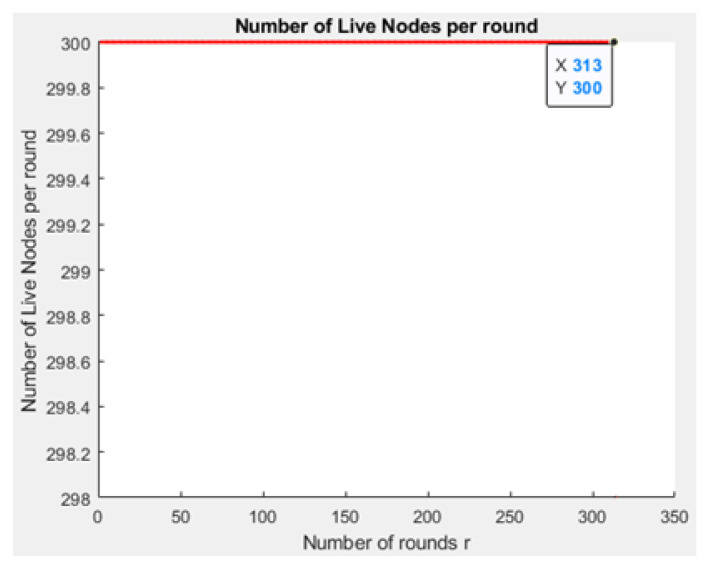
Number of live nodes per round *r* using the LEACH algorithm in NB-IoT D2D attocell network.

**Figure 36:**
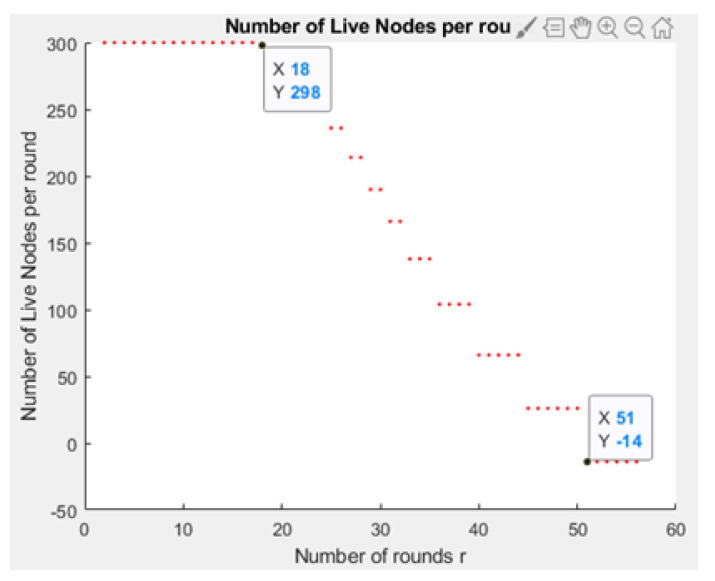
Number of live nodes per round *r* without the LEACH algorithm (i.e., conventional NB-IoT).

**Figure 37:**
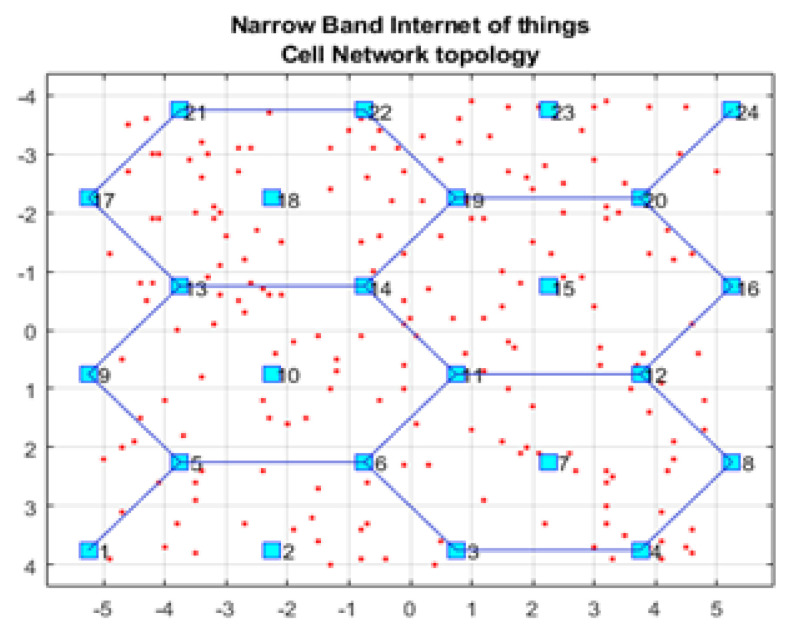
Final result when all nodes die.

Figure 31 and Figure 32 depict the energy dissipation profile of an advanced node and normal node, respectively in the proposed NB-IoT D2D framework, which can be used to determine the SOC and SOH of a device’s battery so they can be included in systems of battery management. This will significantly contribute to reducing overhead costs. Figure 33 depicts the number of transmitted packets in NB-IOT D2D attocell network per round *r*. Figure 34 explains the NB-IoT attocell network throughput (successful received packets rate) using the LEACH algorithm. In other words, the data rate that delivery successfully in the context of LEACH protocol is efficient. Figure 35 and Figure 36 compare the number of live nodes per round *r* in the NB-IoT attocell network using the LEACH algorithm and without using LEACH, respectively. In the presence of the LEACH algorithm (Figure 35), all sensor nodes are live until more than 300 rounds. While without using the LEACH protocol, as shown Figure 36, sensor devices start to die at the round number 18 and at the round number 51, there is no live sensor device in a traditional NB-IoT attocell network. However, all the above measurements are analyzed to present a comprehensive evaluation of the proposed open-sourced simulator. Finally, Figure 37 illustrates NB-IoT attocell network topology when all devices die: In other words, when all nodes have consumed the batteries’ powers entirely.

## 5. Conclusions and Future Work

An NB-IoT network topology in the implementation of the LEACH protocol reduced the queuing delays (latency) at the BS, thus decreasing energy consumption, increasing battery lifetime, minimizing communication cost, permitting better network wrapping, maintaining efficient bandwidth use, and preventing malicious node attacks.

These advantages enhanced the whole network’s lifetime and performance. Moreover, our NB-IoT D2D framework allows researchers and system designers to evaluate different parameters and develop improved NB-IoT designs, increase performance, and reduce the cost of building, operating, and maintaining them since wireless communication of the future will be heavily influenced by the QoS experienced in communication and devices’ expected battery lifetime.

In future, this study could be used to transfer the NB-IoT D2D attocell network to domotic applications in microcells, such as smart homes, and industrial applications to be incorporated into larger systems, such as suburban and urban scenarios involving smart cities, smart grids, smart vehicles, and rural areas comprised of smart farms, as well as the implementation of mobile users’ equipment. This research could also be used to detect different selective malicious node attacks that compromise the routing protocols in the networking layer of NB-IoT systems, study their effects, and use machine learning-powered network communication management. This simulation tool will also contain Simulink models as devices to represent communication features in the physical and application layers, including Excel data sets for managing the input and output variables for analysis purposes. We also hope to improve our simulation to be integral and compatible with new technologies, such as sixth-generation wireless networks. In future, we also intend to develop a cross-layer involving MAC (OFDMA, SC-OFDMA) and LEACH using AI techniques with NB-IoT D2D.

## Figures and Tables

**Table 1:** MATLAB-based Narrowband-Internet of Things (NB-IoT) Device-to-Device (D2D) simulation framework: Key components.

Unit	Main Role	Explanation
NB_IoT_GUI.m	Main code and path loss model	Contains the main functions and the interface user call-backs
NB_IoT_GUI.GUI	Interface	Runs the simulation, shows the network topology and results for path loss models and Signal to Interference plus Noise Ratio (SINR)
LntcyEnrgyPrfrmnc.m	Latency and energy model	Assumes two coverage classes depending on the path loss, thus, generates the matrices for latency and energy
queueCH.m	Cluster Heads (CH) servers’ queuing	Develops the CH queue and determines the total energy consumption for each associated device
queueBS.m	Base Station (BS) servers’ queuing	Develops the BS queue and determines the total energy consumption for each CH

**Table 2:** Parameters of MATLAB-based NB-IoT D2D simulation framework.

Simulation Parameters	Value
maximum number of rounds	999
threshold value: T(n)	design parameter
probability of a node becoming a CH: p	random
packet generation frequency, i.e., one packet per two hours [per day]: S	0.5 × 24
probability of uplink request: $p_{u}$	0.8
network protocol	Low Energy Adaptive Clustering Hierarchy (LEACH)
MAC protocol	OFDMA and SC-FDMA
uplink and downlink frame length moments: $l_{a}$, $m_{a}$	500 bits, 5 kbit
transmission power spectral density: TxSpctrlDnsty	design parameter
penetration loss: TidB	10 dB
average length of RA signaling: τ	0.01 s
average length of control signaling: u	0.002 s
tones	Tones∈[12,6,3,2,1]
bandwidth: B	180 kHz
arrival frequency of control data initiated by BS: $\lambda_{bs}$	1/CF
noise figure: Nsfigr	2.5
noise power spectral density: NsSpctrldnsty	design parameter
devices’ fraction belongs to coverage classes $c_{1}$ and $c_{2}$: $f_{1}$, $f_{2}$	0.5, 0.5
path loss between CHs and BSs: $PL_{CH-BS}$	design parameter dB
path loss between NB-IoT D2D nodes: $PL_{D2D}$	design parameter dB
Signal to Interference plus Noise Ratio: SINR	design parameter dB
distance from CH to BS: d_eNB	design parameter km
repetition order: $c_{1}$, $c_{2}$	1, 2
antenna gain of transmitter: $G_{TX}$	21.10 dB
antenna gain of receiver: $G_{RX}$	19.18 dB
uplink data rate: $R_{1}$, $R_{2}$	5, 5 kbit/s
downlink data rate: ${ℜ}_{1}$, ${ℜ}_{2}$	15, 15 kbit/s
communication model	Bi-direction
synchronization delay: $D_{sy1}$, $D_{sy2}$	0.33, 0.66 s
maximum coupling loss: MCL	144, 155, 164 dB
frame fraction used by reference signals: *b*	0.2
thermal noise spectral density: $N_{0}$	−174 dBm/Hz
greatest waiting time to obtain RAR: $T_{th}$	2 s
number of RA resources: M1,M2	16, 16 preambles
period of time between two NPRACH schedules: *t*	design parameter
period of time between two NPDCCH schedules: td	design parameter
duplex mode	frequency-division duplex
expected energy consumption in uplink class *j* communication: $\xi_{uj}$	design parameter (Joules)
expected energy consumption in downlink class *j* communication: $\xi_{dj}$	design parameter (Joules)
expected battery lifetime: $L_{j}$	design parameter
energy consumption in listening: $E_{sy}$	Joules
energy consumption in RAR message: $E_{rar}$, $E_{rr}$	Joules
initial energy for a node: Eo	1000 Joules
interference: *I*	0.50
power spending in sending, idle, and listening: $P_{t}$, $P_{I}$, $P_{l}$	0.2, 0.01, 0,1 Watts
distance from each node in the cluster to the closest cluster head CH per round r: $d_{CH-N}$	design parameter km
power spending in electronic circuits: Pc	0.01 Watts
transmission power: $PTX_{j}$	14 dBm

## Data Availability

The source codes of MATLAB-based NB-IoT D2D simulation framework are available on the MDPI website as supplementary material and on GitHub (https://github.com/Ohood-Althobaiti/NB-IoTD2DSimulation-AnOpen-SourcedFramework).

## Supplementary Materials

Supplementary Materials are available online at https://www.mdpi.com/1424-8220/21/5/1824/s1.
